# Scratch-Healing Behavior of Ice by Local Sublimation and Condensation

**DOI:** 10.1021/acs.jpcc.1c09590

**Published:** 2022-01-19

**Authors:** Menno Demmenie, Paul Kolpakov, Yuki Nagata, Sander Woutersen, Daniel Bonn

**Affiliations:** † Institute of Physics, University of Amsterdam, Science Park 904, 1098 XH Amsterdam, The Netherlands; ‡ Van’t Hoff Institute for Molecular Sciences, University of Amsterdam, Science Park 904, 1098 XH Amsterdam, The Netherlands; § Max Planck Institute for Polymer Research, Ackermannweg 10, 55128 Mainz, Germany

## Abstract

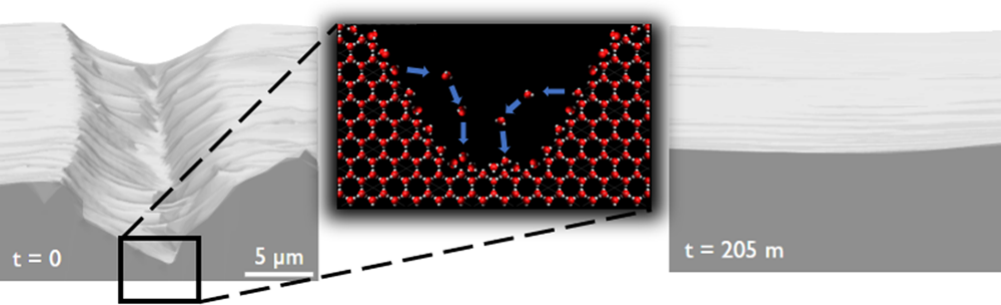

We show that the surface of ice is scratch healing: micrometer-deep scratches in the ice surface spontaneously disappear by thermal relaxation on the time scale of roughly an hour. Following the dynamics and comparing it to different mass transfer mechanisms, we find that sublimation from and condensation onto the ice surface is the dominant scratch-healing mechanism. The scratch-healing kinetics shows a strong temperature dependence, following an Arrhenius behavior with an activation energy of Δ*E* = 58.6 ± 4.6 kJ/mol, agreeing with the proposed sublimation mechanism and at odds with surface diffusion or fluid flow or evaporation–condensation from a quasi-liquid layer.

## Introduction

Ice is one of the most actively studied solids,^1−11^ and many of its physical properties are still poorly understood. In particular, the structure and dynamics of the topmost layer of water molecules in ice has been the subject of intense debate for more than 150 years. Already in 1860, Faraday observed that ice cubes sinter together, and he concluded that there is always a liquid layer present on the ice surface, even at atmospheric temperatures far below the melting temperature.^12,13^ It took more than 100 years until detailed measurements of the speed of sintering finally ruled out the idea that the flow of a liquid layer was at the origin of the sintering dynamics.^14−19^ Explanations of the mass transfer phenomenon underlying ice sintering included highly mobile surface molecules undergoing surface diffusion,^16^ bulk lattice motion,^20^ and condensation from the vapor phase.^21−23^ Although these three forms of thermal relaxation were studied thoroughly in the 1950s and 1960s, the mechanism that dominates the sintering process of ice has never been quantitatively established. Hence, Kingery’s explanation by surface diffusion is frequently considered as being correct, even though it yields an activation energy of more than twice the latent heat of sublimation.

More recent research suggests that the outermost molecular layer of an ice crystal is disordered.^24−28^ However, this one to two molecules thick layer cannot simply be considered as a liquid since it exhibits viscoelastic properties.^29,30^ General crystal growth theory is insufficient to describe the diffusion limited dynamics of ice crystals since it does not account for the ambiguous disordered interface, cooperative intermolecular hydrogen bonding, and the degree of supercooling. Moreover, no model has been proposed that completely describes the unique growth behavior of ice thus far.^31−35^

To shed new light on the complex molecular dynamics of the surface of ice, we investigate the temporal evolution of a scratch made in a pristine surface of ice with submicrometer precision, under precisely controlled experimental conditions. We find that the scratch heals spontaneously over time, and that eventually the ice surface becomes completely smooth again. By comparing the data quantitatively to models for the different proposed healing mechanisms, we determine which mechanism dominates the healing process. We conclude that scratch healing of ice occurs by the detachment and reattachment of surface molecules. Since the transport of water molecules in the ambient phase is limited by diffusion, this process is dominated by local sublimation from and condensation onto the surface.^36^ The obtained activation energy corresponds to the known value for sublimation, which is significantly higher than the energy barrier for liquid evaporation. This settles the long-standing debate on the origin of the sintering dynamics of ice and explains why healing occurs relatively quickly in ice with its high vapor pressure.

## Methods

Measurements were carried out with a confocal profilometer (Keyence VK-X1100), with a lateral resolution of 212 nm and a vertical resolution of 12 nm,^37^ in a temperature and humidity controlled chamber. The humidity was regulated by an inflow of dry nitrogen into the chamber and monitored by a thermal hygrometer (Testo 645, error of 0.1% relative humidity). Furthermore, to reach low humidities, a controlled flow of liquid nitrogen (Norhof Microdosing LN2, 900 series) through a copper element acted as a cold trap to remove remaining water vapor by condensation. Hence, a theoretical equilibrium between the vapor pressure of the flat ice surface and the air could be achieved, which was calculated with the parameters of Murphy and Koop.^38^ When the measured humidity deviated from the theoretical equilibrium by a larger value than the error margin of the hygrometer, the inflow of nitrogen was adjusted. Apart from the healing of scratches, no sign of sublimation or condensation was observed on the horizontal surfaces, confirming the stability of the equilibrium in the chamber.

The ice layers were formed by cooling 3 mL of ultrapurified water from a Milli-Q system on a copper plate (560 × 380 × 40 mm). Cooling was done by a Peltier element in direct contact (using thermal paste) with the copper plate to ensure an isothermal and homogeneous layer of ice. The induced heat on the opposite side of the Peltier element was extracted from the system by flow from a temperature bath. Ice temperatures were in the range from 243.0 to 272.6 K (measured by a Voltcraft PL-125-T2USB VS temperature probe). Micrometer-sized scratches were manually created with a sharp razor blade (Derby extra Paslanmaz Çelik) and positioned in such a way that the measured area of 212 × 283 μm contained only one defined crystal orientation, so grain-boundary dynamics could be excluded. Since different grains do not exhibit wide variations in molecular organization at the surface, we were enabled to collect an ensemble of measurements performed under similar experimental conditions, as the effective diffusion coefficients are expected to be similar.^39^ The profile of the ice was regularly monitored by a 50× magnification Plan Apo objective (NA 0.95, WD 0.35 mm, 404 nm wavelength reflection) for the period of scratch healing. For a detailed 3D model of the setup, see Figure S1.

## Results and Discussion

**Figure 1:**
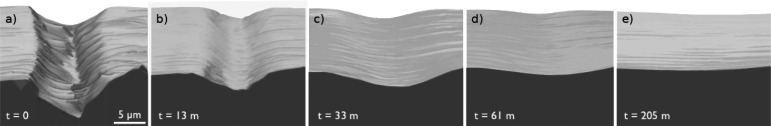
Evolution of a scratch in ice (initial depth ∼2.5 μm) healing in time under controlled conditions, with constant ice temperature of 247 K and vapor pressure at equilibrium.

**Figure 2:**
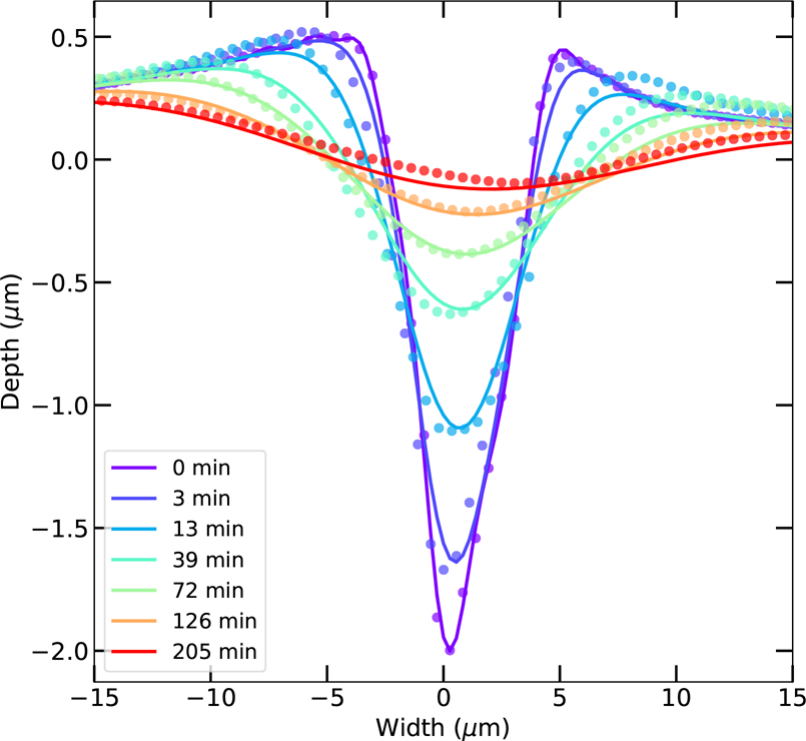
Self-healing of a micrometer-sized scratch in ice (*T*_ice_ = 247 K). For each time step, dots depict data taken by profilometry, whereas solid lines are fits by the sublimation–condensation model. For clarity, seven time steps are shown of the 22 recorded in total.

Our measurements provide highly detailed images of the ice scratch profile as it slowly heals in time. As illustrated in Figure 1, the initially sharp-edged scratch evolves into a smooth profile and eventually disappears altogether. To quantify these dynamics and to avoid measuring local impurities in the ice, we average scratch cross sections over a length of 220 μm; see Figure 2 (solid points). The resulting data allow us to experimentally test the four ice-healing mechanisms proposed thus far: (1) a fluid flow of liquidlike water molecules from the outermost layer, (2) displacement by local sublimation and condensation, (3) movement by volume diffusion as a bulk process, and (4) a rearrangement of the topmost loosely bound molecules by surface diffusion. To this end, we numerically solve the differential equation of each model and compare the results to the experimentally observed time-dependent scratch profile.

The theoretical basis for each of the four mechanisms was given for the one-dimensional case by Mullins.^40,41^ The Mullins model assumes that the attachment and detachment of molecules can occur everywhere on the surface, which is valid for ice with its disordered interface. He derived that, in the case of an initial sinusoidal profile with wavelength λ, only the overall amplitude of the profile changes with time, so the time-dependent distance of the ice surface with respect to the unscratched surface is given by *U*(*x*, *t*) = *u*(*t*) sin(2π*x*/λ), where *x* is the direction perpendicular to the scratch and *t* is time. The evolution equation for the amplitude *u*(*t*) depends on the mechanism and is given by
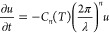
1where *C*_*n*_(*T*) is a temperature-dependent prefactor and *n* is an integer depending on the model: *n* = 1 for fluid flow, *n* = 2 for sublimation–condensation, *n* = 3 for volume diffusion, and *n* = 4 for surface diffusion. Mullins also showed that the equations governing the mass diffusion are linear in the sense that the sum of any two solutions is again a solution.^41^ Hence, the evolution of an arbitrary initial profile can be obtained from a Fourier analysis, and this is how we calculate the time-dependent profiles for each of the four models: we decompose the initial experimental profile as a Fourier sum (using the 90 lowest-spatial-frequency Fourier components) and propagate each component independently in time. We apply a correction for a small overall slope of the initial profile if necessary. Our analysis involves the following simplifying assumptions: (i) the measurements are carried out in a closed system where the vapor pressures of the flat ice surface and air are in equilibrium, (ii) the mass transfer coefficients are not affected by the crystal orientation of the ice lattice, and (iii) the slope of the profiles is small enough to apply the small-slope approximation ( ≪ 1).

**Figure 3:**
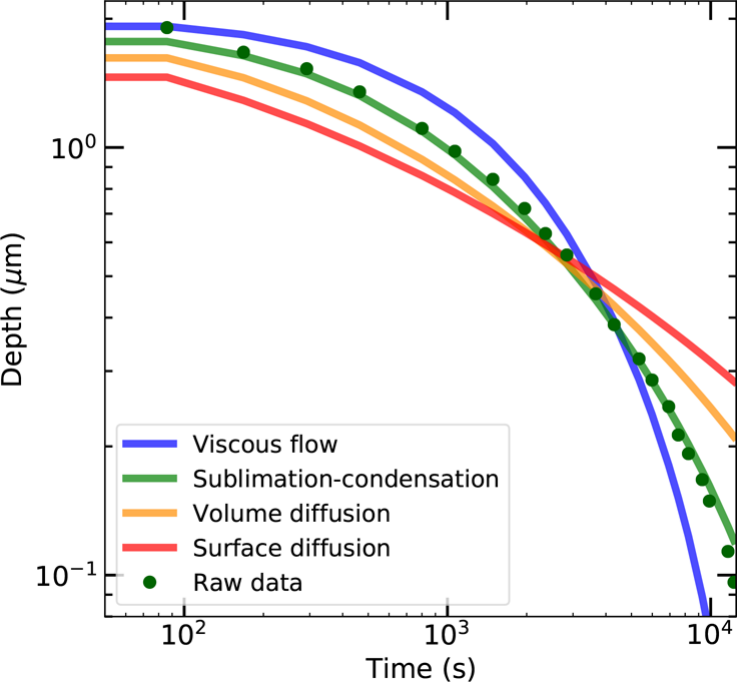
Maximum depth of an ice scratch as a function of time. Green dots indicate experimental data; solid lines indicate best fits of the four different candidate models.

We test each mass transfer model by comparing the theoretical prediction with the data of 30 independent measurements using χ^2^ minimization, with *C*_*n*_(*T*) as the only free parameter. We find that the sublimation–condensation process exhibits the lowest χ^2^ and thus best agreement with the measurements (Figure S2). In Figure 2, we show this agreement for the scratch of Figure 1; the least-squares fits to the other models are shown in Figure S3. The differences among the four models are further illustrated by plotting the absolute maximum depth of the ice scratch profile developing in time for the best fitting parameters *C*_*n*_(*T*) in Figure 3. Clearly, the best description of the dynamics is given by the sublimation–condensation model.

In the sublimation–condensation driven healing process, the smoothening of the profile is driven by an increased vapor pressure for curved surfaces (i.e., the Kelvin equation; for the full derivation see the Supporting Information). This scratch-healing process is relatively fast in the first few minutes and slows down as the surface becomes less curved, as observed in Figure 3.

**Figure 4:**
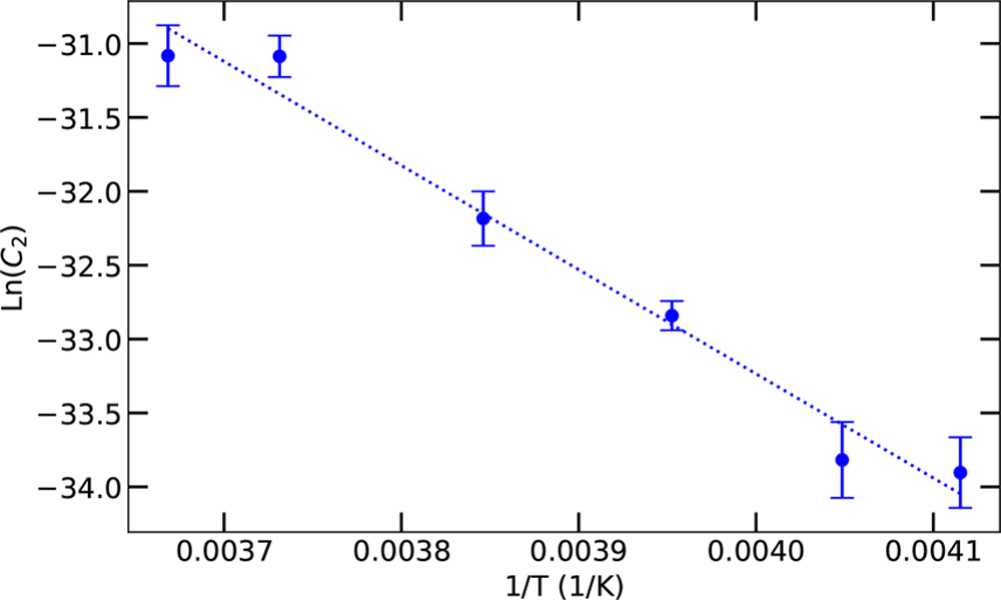
Arrhenius behavior of the sublimation–condensation coefficient *C*_2_. Each data point in blue represents five measurements on five different scratches; a linear fit yields an activation energy of Δ*E* = 58.6 ± 4.6 kJ/mol.

To obtain more insight into the healing mechanism, we investigate the temperature dependence of the effective diffusion coefficient. To this end we perform similar least-squares fit analyses for 30 measurements (five scratches profiled at six different temperatures from 243.0 to 272.6 K). The effective sublimation–condensation coefficients *C*_2_(*T*) obtained from the fit follow an Arrhenius temperature dependence with an activation energy of Δ*E* = 58.6 ± 4.6 kJ/mol, as shown in Figure 4. For comparison, the latent heat for the sublimation of water molecules is approximately 51.1 kJ/mol.^38^ The sublimation activation energy of water was found to be in the range 53.1–57.3 kJ/mol,^42−44^ in good agreement with our result. Moreover, the energy barrier for condensation is significantly lower: 43.35–45.1 kJ/mol.^45,46^ These results are strong indications that the scratch healing of ice is driven by local sublimation instead of local evaporation, before condensation occurs.

The above results indicate that the scratch healing of ice occurs through a sublimation–condensation mechanism. We now discuss earlier experimental results that were previously interpreted in terms of the other proposed scratch-healing mechanisms. First, consider the liquid layer interpretation of Faraday. This concept received widespread acclaim by rather precarious comparisons between the physical properties of thin liquid layers and the topmost layer of ice.^47−49^ However, none of the sintering experiments could be quantitatively reproduced by the liquid layer model.^3^

Second, the research that concluded that volumetric bulk diffusion is responsible for the sintering of small ice beads was conducted in a liquid kerosene saturated environment.^20^ This inhibited the movement of the molecules along the surface and, more importantly, completely prevented mass diffusion via the vapor phase. Hence, this specific experiment on ice sintering is not generally applicable.

Third, the research on surface diffusion, done by Kingery, matched the theoretical predictions of the neck growth between two touching ice spheres. However, the obtained activation energy of approximately 115 kJ/mol is more than twice the latent heat of sublimation.^16^ Remarkably, when we force the model for surface diffusion onto the data of our scratch-healing experiments, we obtain a similar activation energy of Δ*E* = 100.0 ± 11.1 kJ/mol. Weber et al. used friction experiments and molecular dynamic simulations on the topmost layer of solid water molecules to demonstrate that the activation energy of surface diffusion is roughly 11.5 kJ/mol: 1 order of magnitude lower.^50^

## Conclusion

To conclude, we find that the detachment and reattachment of highly mobile water molecules on the ice surface causes scratches in the ice surface to heal spontaneously. By quantitatively studying the scratch-healing behavior of micrometer-sized scratches, and comparing the results with four models proposed for the transport of molecules on the ice surface, we conclude that the main mechanism of transport is through sublimation and condensation. We propose that the efficient scratch healing of ice compared to other materials might be due to the water molecules in ice being connected by hydrogen bonds: in contrast to the attractive interactions in the crystals of most other materials, hydrogen bonding is highly cooperative, meaning that breaking four hydrogen bonds in the bulk requires much more than 2 times the energy required for breaking two hydrogen bonds at the interface. As a consequence, the water molecules at the surface can detach relatively easily, even though the bulk crystal phase is completely stable.

## Supporting Information Available

Additional details on the derivation of the dominant model, experimental setup, and statistical comparison between healing mechanisms (PDF)
