# Supplementary material for: Scratch-Healing Behavior of Ice by Local Sublimation and Condensation
Source: J Phys Chem C Nanomater Interfaces. 2022 Jan 19;126(4):2179–83. doi: 10.1021/acs.jpcc.1c09590 (PMC8819648; doi:10.1021/acs.jpcc.1c09590)
Supplement: Supplementary file 1 — jp1c09590_si_001.pdf [file jp1c09590_si_001.pdf]

Scratch-Healing Behavior of Ice by Local Sublimation and Condensation

Menno Demmenie,*,†,‡ Paul Kolpakov,† Yuki Nagata,¶ Sander Woutersen,‡ and Daniel Bonn†

†*Institute of Physics, University of Amsterdam, Science Park 904, 1098 XH Amsterdam, the Netherlands*

‡*Van 't Hoff Institute for Molecular Sciences, University of Amsterdam, Science Park 904, 1098XH Amsterdam, The Netherlands*

¶*Max Planck Institute for Polymer Research, Ackermannweg 10, 55128 Mainz, Germany*

E-mail: m.demmenie@uva.nl

Supporting Information

For the derivation of sublimation-condensation model (n = 2), we start with the Kelvin equation, showing the relation between the vapor pressure of a surface with respect to a curved surface with radius r:

$$\ln\left(\frac{P}{P_{sat}}\right) = \frac{2\gamma V_m}{rRT}, \tag{1}$$

where P and P_{sat} are the vapor pressures of the curved and flat surface, respectively; γ is the surface tension, V_m the molar volume, R the universal gas constant, and T the temperature. Using the definition of curvature $\kappa = 1/r$, we can define the Kelvin equation along the curve of the scratch:

$$\ln\left(\frac{P}{P_{sat}}\right) = \frac{2\kappa\gamma V_m}{RT} \tag{2}$$

Since we are measuring relatively small curvatures we can use the first order Taylor expansion for $ln(P/P_{sat})$ around $\ln(1) = (P/P_{sat} - 1)$ and call $P - P_{sat} = \Delta P$. Then we obtain:

$$\frac{\Delta P}{P_{sat}} = \frac{2\kappa\gamma V_m}{RT} \tag{3}$$

Classic kinetic theory of gasses states that the number of emitted atoms per surface area per second $\Delta\theta$ is given by:

$$\Delta\theta = \frac{\Delta P}{\sqrt{2\pi MRT}}, \tag{4}$$

with M the molecular weight. Note that we omit an extra Arrhenius factor on the right-hand side, that usually represents the attachment or detachment process in crystal growth theory. This term is negligible in the case of ice crystals since the limiting process is the diffusion of water molecules in the vapor phase. Filling in ΔP from Eq. 4, and multiplying both sides

with V_m yields the velocity of the volume per unit area of emitted particles:

$$V_m \Delta\theta = \sqrt{\frac{2}{\pi M}} \frac{P_{sat} \kappa \gamma V_m^2}{(RT)^{\frac{3}{2}}} \tag{5}$$

The curvature κ can be denoted as:

$$\kappa = \frac{\partial^2 y}{\partial x^2} \left(1 + \left(\frac{\partial y}{\partial x} \right)^2 \right)^{-\frac{3}{2}}, \tag{6}$$

and the projected velocity in the y-direction of $V_m \Delta\theta$ as:

$$V_m \Delta\theta = \sqrt{1 + \left(\frac{\partial y}{\partial x} \right)^2} \frac{\partial y}{\partial t} \tag{7}$$

Together with the small slope approximation $\frac{\partial y}{\partial x} \ll 1$, we obtain:

$$\frac{\partial y(x,t)}{\partial t} = C_2(T) \frac{\partial^2 y(x,t)}{\partial x^2}, \tag{8}$$

which results, for a sinusoidal case, in:

$$\frac{\partial y(x,t)}{\partial t} = -C_2(T) k^2 y(x,t), \tag{9}$$

with $C_2(T) = \sqrt{2} P_{sat} \gamma V_m^2 / ((\pi M)^{\frac{1}{2}} (RT)^{\frac{2}{3}})$ and $k = \frac{2\pi}{\lambda}$.

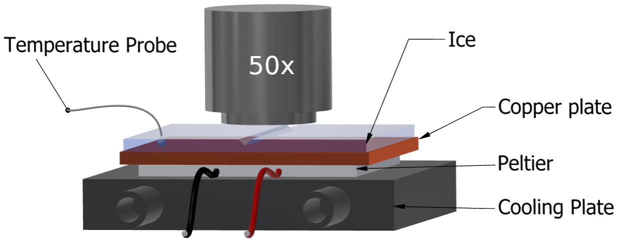

Figure S1: Schematic view of the part of the experimental setup that was placed on a motorized stage with sub-micron lateral precision. This system was placed in a temperature and humidity controlled chamber.

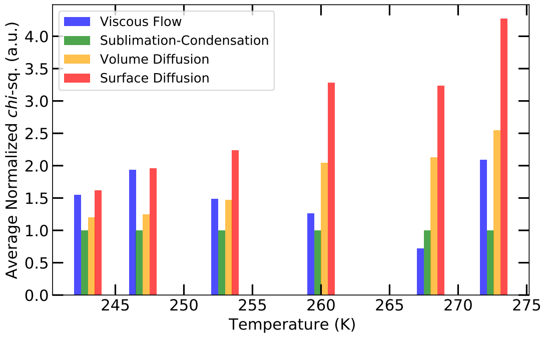

Figure S2: χ-square quantification of the best fits of each of the 4 models to the experimental data at 6 temperatures. For each temperature we averaged 5 measurements that were normalized to the χ-square value of the sublimation-condensation model.

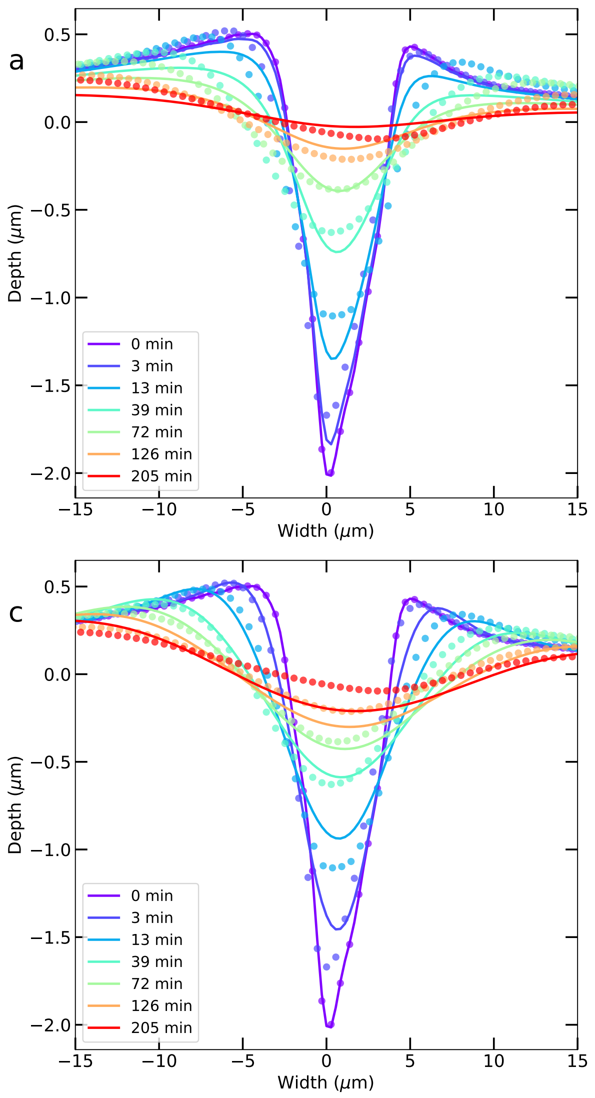

Figure S3: Comparison between the four models for the scratch-healing of a micron-sized scratch in ice ($T_{\text{ice}} = 247$ K). For each time step, dots depict data taken by profilometry, whereas solid lines are fits by the viscous flow (a), sublimation and condensataion (b), volumetric bulk diffusion (c) and surface diffusion (d) model. For clarity, seven time steps are shown of the 22 recorded.
